# Book Reviews

**Published:** 1904-02

**Authors:** 


### BOOK REVIEW.

Success Without Money.—The brisk attack on snobbery that was a leading feature of the October *Everybody's* has been followed up in the November issue by a significant article on "Successful Men who are not Rich." Success without money seems anomalous to the modern American: yet, on reflection, the most eager devotee of plutocracy cannot deny that such men as Senator Hoar of Massachusetts, President Eliot of Harvard, Dr. Rainsford and General Joe Wheeler are, in the best sense of the term, successful. And the private income of the most successful man in the United States to-day—Theodore Roosevelt—is said to be well under $7,500 yearly. It is good to be reminded of these triumphs that are not measured in dollars.

---

The Youth's Companion in 1904.—As the years increase the youth's companion endeavors to keep pace with them in all that is wise, beautiful and progressive, and not only to retain but to deserve the honorable and exceptionally high place it holds in the confidence and affection of three generations of readers. The greatest living authors in all branches of literature continue to contribute to it.

Among the important series of articles will be one on the occupation of the farmer in many parts of the world—in England, in Ireland, in India, in Argentina, etc.

The annual Announcement Number of THE COMPANION, describing the principal features of THE COMPANION'S new volume, will be sent to any address, Free.

The new subscriber for 1904 will receive all the issues of THE COMPANION for the remaining weeks of 1903 free from the time of subscription; also THE COMPANION Calendar for 1904, lithographed in twelve colors and gold.—THE YOUTH'S COMPANION, 144 Berkeley Street, Boston, Mass.

---

THE EDUCATION OF BOYS.—In *The Delineator* for *November*, Mrs. Theodore W. Birney has a suggestive paper on the Education of Boys as future Fathers and Citizens. The gist of her argument is that boys seldom receive the sympathy to which they are entitled—not a maudlin, sentimental sympathy that is calculated to spoil the child, but an intelligent comprehension of his needs and an interest in his doings and belongings. Her conclusion is that if parents will only take a genuine interest in all things that interest their boys, they can hold their confidence, and so long as they possess that they can be reasonably sure that their sons will not go far wrong. Parents are wont to look on the problems of youth with the eyes of an adult. How much good would result to many little fellows if their parents would come down to their view point, or come up perhaps, recognizing the limitations of their inexperience, and judging their deeds and misdeeds in the light of it. The rule of the rod is past, and inasmuch as the new order of things has brought much happiness into the lives of the little ones, so will a better understanding of the boy nature on the part of parents benefit them immeasurably.

Porcelain a Preserver of the Pulp.—Dr. Head spoke of porcelain as a preserver of pulp. I could cite numerous instances in which I had complete exposure of pulps, many of them by accident, and in which the pulp was in a healthy condition. After putting in inlays there has been no further trouble. About nine months ago a young girl was brought to me who had broken one of her central incisors, leaving an exposure of the pulp. I washed the cavity out with carbolic acid and put a little temporary filling upon the tooth. The tooth was sensitive until the next appointment. I made an inlay, and knowing where the pulp was exposed, I avoided burnishing over that spot. When the inlay was made and set she experienced no pain from the pressure, but a little from the heat of crystallization. In fifteen minutes after it was set she was entirely free from all pain, and has never had the slightest discomfort from the tooth since.—International Dental Journal.

---

Matching Colors in Inlay Work.—It does not matter how near one comes in matching the tooth, the natural translucent effect so necessary cannot be obtained when a solid color is used. Every tooth has more than one color or shade, which can best be described by calling them underlying colors. A tooth that at first sight appears to be just one color will, when closely examined, be seen to have two or three or more underlying colors, and to get the effect we are striving after, these colors must appear in the filling. This can only be done by baking the different colors in layers. It may seem almost impossible at first to see these different colors of the teeth, but you will be surprised when you have practiced looking for them how in a short time you will begin to detect them easily.—Dental Register.

---

To induce solder to flow in a hole or space, pack gold foil in the hole or space, borax the part and the solder will fill the cavity perfectly.

